# Pylorus resection or pylorus preservation in partial pancreatico-duodenectomy (PROPP study): study protocol for a randomized controlled trial

**DOI:** 10.1186/1745-6215-14-44

**Published:** 2013-02-14

**Authors:** Thilo Hackert, Thomas Bruckner, Colette Dörr-Harim, Markus K Diener, Phillip Knebel, Werner Hartwig, Oliver Strobel, Stefan Fritz, Lutz Schneider, Jens Werner, Markus W Büchler

**Affiliations:** 1 Department of General, Visceral and Transplantation Surgery, University of Heidelberg, Im Neuenheimer Feld 110, Heidelberg, 69120, Germany; 2 Institute of Medical Biometry and Informatics, University of Heidelberg, Im Neuenheimer Feld 305, Heidelberg, 69120, Germany; 3 Study Centre of the German Surgical Society and Department of General, Visceral and Transplantation Surgery, University of Heidelberg, Im Neuenheimer Feld 110, Heidelberg, 69120, Germany; 4 Department of Surgery, University of Heidelberg, Im Neuenheimer Feld 110, Heidelberg, 69120, Germany

**Keywords:** Partial pancreatico-duodenectomy, Pylorus preservation, Pylorus resection, Randomized trial

## Abstract

**Background:**

Partial pancreatico-duodenectomy (PD) is the standard treatment for tumors of the pancreatic head. Today, preservation of the pylorus has been widely accepted as the surgical standard in this procedure. A common postoperative complication is the occurrence of delayed gastric emptying (DGE), which causes impairment of oral intake andpatients’ quality of life, prolongation of hospital stay and delay of further treatment (for example adjuvant chemotherapy). In a small number of two retrospective and one randomized studies, a modification by resection of the pylorus with preservation of the stomach has shown to reduce DGE incidence. The aim of the present study is to investigate the effect of pylorus resection on postoperative DGE in PD.

**Methods/Design:**

Patients undergoing elective PD for any indication equal or older than 18 years and who have given informed consent will be included. Patients will be randomized to either PD with pylorus preservation or PD with pylorus resection and complete stomach preservation. Sample size (n = 89 patients per group) is calculated on an assumed difference in DGE incidence of 20%. Primary study endpoint is DGE within 30 days; secondary endpoints are operation time, blood loss, morbidity, mortality, hospital stay and quality of life (QoL).

**Discussion:**

DGE is a relevant clinical problem following PD with a great impact on patients’ recovery, length of hospital stay, QoL and consecutive adjuvant therapies. As there is no causal therapy, prevention of DGE is essential to improve outcome. The technical modification of pylorus resection may offer a simple and effective method for this purpose. The present study is designed to increase the existing body of evidence and potentially change the future standard surgical procedure of PD.

**Trial registration:**

German Clinical Trials Register DRKS00004191.

## Background

Partial pancreatico-duodenectomy (PD) is the standard treatment for tumors of the pancreatic head as well as benign precursor lesions such as intraductal mucinous neoplasia (IPMN) that require a resective surgical approach [1,2]. Classical partial pancreatico-duodenectomy with resection of the distal stomach as the historical standard procedure was modified in the 1970s by Traverso, who introduced preservation of the pylorus [3]. This modification (ppPD) has been widely accepted to be equally effective compared to the classical pancreatico-duodenectomy with regard to tumor recurrence and long-term survival in numerous studies [4]. A well-known complication after either method of PD is the occurrence of delayed gastric emptying (DGE) [5,6]. DGE causes impairment of oral intake and patients’ quality of life (QoL), prolongation of hospital stay and delay of further relevant treatments (for example start of adjuvant chemotherapy). This complication is regarded as a functional impairment of the physiological propulsive regulation of the stomach and especially the pylorus. In recent trials, it has been shown that antecolic reconstruction is superior to retrocolic duodeno-jejunostomy in terms of the incidence of DGE [5,6]. In 2007, the International Study Group of Pancreatic Surgery (ISGPS) proposed a standardized definition of DGE with three grades of severity (A-C) [7]. The grading system included the use of a gastric tube, time to solid oral food intake, symptoms like distension and vomiting as well as the use of prokinetic medication. This definition was evaluated in the patient collective in Heidelberg [8]. The overall frequency of DGE within this collective was 45%, including 28% DGE grade A, 8% grade B and 9% grade C.

In a recent study published in 2010 by Kurahara *et al*. [9], the frequency of DGE using the ISGPS definition was examined in a retrospective data analysis comparing 48 patients that underwent ppPD vs. 64 patients in which the pylorus was resected (prPD). Resection was limited to the pyloric ring with preservation of the entire stomach. Reconstruction was then performed as a gastro-jejunostomy in the distal antrum of the stomach. In this study, DGE frequency was 34.8% after pylorus preservation vs. 13.0% when the pylorus was resected. This is in line with a retrospective study published in 2000 [10] comparing similar approached in 39 (ppPD) vs. 33 (prPD) patients. DGE frequency was 33% vs. 12% confirming an approximate difference of 20%. A current Japanese publication by Kawai *et al*. [11] has focused on the issue of pylorus resection in a randomized controlled study. The authors have included 64 patients with ppPD vs. 66 patients that underwent prPD with the endpoint DGE between 7 days and 6 months postoperatively. The observed frequency of DGE was 17.2% and 4.5% respectively (*P* = 0.02). Although this study showed a clear trend toward the results observed in the former retrospective studies, several shortcomings (only one randomized controlled trial (RCT), other studies performed retrospectively, overall small number of patients) do not allow drawing a final conclusion and the surgical consequences. From the mentioned studies, it seems reasonable that resection of the pylorus may offer significant benefits in the postoperative phase regarding the frequency of DGE and consecutive quality of life and may be superior to the standard ppPD. Yet, scientific evidence to support this hypothesis is rather weak.

Therefore, the aim of the present study is to investigate the two modifications of PD (ppPD vs. prPD) in a randomized controlled setting to confirm or contradict the hypothesis and show potential shortcomings of both methods, which are not yet obvious. The results can add more evidence to the current discussion and have a potential impact on a widely performed standard operation in pancreatic surgery.

## Methods

### Study population

The study population consists of all patients scheduled for PD for any indication. Further inclusion criteria are age equal or older than 18 years and having given informed consent. Participation in another intervention trial with interference of intervention and/or outcome of this study and expected lack of compliance as well as language problems represent the only exclusion criteria. The study protocol has been approved by the local ethics committee (University of Heidelberg S-121/2012).

### Randomization and surgical procedures

**Figure 1:**
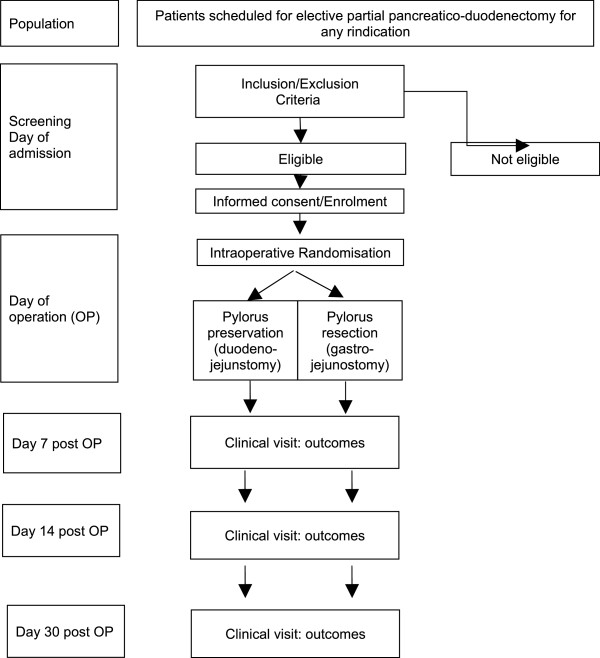
Flow chart of the PROPP study.

Patients are intraoperatively randomized to either of the two groups after the surgeon has confirmed that preservation of the pylorus is technically and oncologically possible. Randomization is performed as an unstratified block randomization with fixed block sizes in a 1:1 allocation ratio using established randomization software. Block size is kept confidential until completion of recruitment. Standard PD with preservation of the pylorus is performed by dividing the duodenum 2 cm distal of the pylorus with a linear stapling device under preservation of the gastric vessels along the lesser and the greater curvature. Antecolic duodeno-jejunostomy is performed at the end of the operation approximately 50 cm distal to the hepato-jejunostomy by an end-to-side anastomosis using two-layer monofilament atraumatic running sutures. Resection of the pylorus is performed with preservation of the stomach by the use of a linear stapling device with complete preservation of the gastric vessels along both curvatures to preserve perfusion of the distal stomach via the gastroepiploic arcade and the left gastric artery, respectively. Antecolic gastro-enterostomy is performed by an end-to-side gastro-jejunostomy using a two-layer running monofilament atraumatic suture technique as well. In both groups, nasogastric tubes are removed as soon as mechanical ventilation is stopped, usually at the end of the operation. Follow-up examinations are scheduled at day 7, 14 and 30 postoperatively. The study protocol is shown in Figure 1.

### Study objectives

Primary efficacy endpoint is the frequency of postoperative DGE according to the international ISGPS definition (Table 1) 30 days after index operation, compared between the two intervention groups [7]. Key secondary endpoints are operation time (skin cut to last knot), blood loss (ml), overall morbidity according to the Clavien-Dindo classification [12], 30-day mortality, postoperative hospital stay and QoL on postoperative day 30.

**Table 1:** **DGE (grading A-C) according to the ISGPS definition**[7]

**Grade**	**Nasogastric tube**	**Solid food intake**	**Vomiting distension**	**Prokinetic medication**
**A**	4-7d/reinsertion > d3	d7	±	±
**B**	8-14d/reinsertion > d7	d14	+	+
**C**	>14d/reinsertion > d14	d21	+	+

DGE, delayed gastric emptying; ISGPS, International Study Group for Pancreatic Surgery.

### Methods against bias

#### Selection bias

Consecutively screened and eligible patients will be included after initiation of the study. In order to achieve comparable intervention groups, patients will be allocated concealed by intraoperative randomization at the day of surgery using a centralized web-based tool ( http:// randomizer.at). Block randomization will be performed to achieve totally equal group sizes. A sufficient number of patients will be recruited according to the sample size calculation in order to prevent random error and to achieve sufficient power for hypothesis testing of the primary endpoint.

#### Information bias

All surgeons who participate in this trial will be instructed which treatment procedures are applicable in both groups. Blinding of surgeons is not feasible due to the nature of the interventions. However, patient and observer documenting the endpoints during the postoperative visits will be blinded.

#### Confounding

Potential confounding will be controlled due to randomization.

Performance bias will be minimized by applying a standardized treatment manual for the surgical as well as the perioperative procedures. Thus, all surgical procedures will be defined in operation manuals. Moreover, medical treatment including prokinetic drugs, proton pump inhibition, antibiotics and octreotide analogs will be defined *a priori*. Routine management of nasogastric tubes and endoscopic examination in case of DGE will be defined as well. Blinded assessment of the primary endpoint 30 days after the surgical procedure will be performed by independent observers (not aware of the respective surgical procedure) to minimize measurement bias. Patients will be kept blind to the randomized and performed procedure. Unblinding of the patients will be possible in situations requiring reinterventions.

### Sample size calculation

Sample size calculation is based on the primary endpoint ‘frequency of DGE 30 days after surgery’. On the basis of previous literature mentioned in the background section, an assumed absolute risk of 20% difference in DGE frequency is the basis for the calculation assuming 32% DGE in group 1 (ppPD) and 12% in group 2 (prPD).This leads to a calculated number of 89 patients who have to be treated in each group assuring a power of 90% at a two-sided level of significance of 5%. As the individual results for the primary endpoint are available within 30 days after surgery, the expected drop-out rate is small. Nevertheless, a potential dilution of the treatment effect due to drop-outs is taken into account; it is assumed that this can be compensated by additional 10% of patients to be randomized, and therefore the total sample size required amounts to n = 198 patients.

### Documentation and data handling

All protocol-required information collected during the trial is entered in the case report form (CRF). The completed CRFs are reviewed and signed by the investigator or by a designated sub-investigator and sent to the Institute for Medical Biometrics and Informatics (IMBI) for data entry. The data will be managed and analyzed in the joint unit of the clinical study center and IMBI in accordance with the appropriate standard operating procedures (SOPs) valid in the IMBI. A safety analysis will be based on the set of all patients for which one of the interventions was applied. Serious adverse events will be tabulated, absolute and relative frequency, severity and the relationship to the intervention will be given and compared between the intervention groups.

## Discussion

DGE is a common complication after PD reported in numerous studies, initially under the use of various definitions [5,6]. Consequently, highly differing rates of DGE were reported, which led to the introduction of the standardized definition of the ISGPS in 2007 [7]. The proposed definition has been evaluated clinically in large patient groups after ppPD and surprisingly shown an actual overall DGE incidence of up to 45% [8].

DGE represents a therapeutically difficult complication that prolongs patients’ hospital stay and postpones or even inhibits the start of adjuvant chemotherapy in tumor patients. Therefore, the need to reduce the frequency of DGE is obvious. In two retrospective studies a 20% reduction of DGE after prPD was shown [9,10]. A current randomized trial [11] confirmed a reduction of DGE although the 13.7% difference between prPD and ppPD in this study was less pronounced. As the number of patients in these available studies is limited and the study designs are heterogenous, evidence is still weak and further studies are necessary to evaluate the actual impact of prPD on DGE.

It remains unclear if pylorus resection influences the severity of DGE in terms of the ISGPS grading A-C. Furthermore, no standardized therapy of DGE is defined yet, which impairs the significance of the respective results. Reconstruction technique with stenting of the pancreatic duct in the RCT [11] cannot be considered as the standard reconstruction. As the overall fistula rate was rather high in this study, this could have a substantial influence on DGE. Postoperative nasogastric tube management is not standardized. From our experience, tube removal can be routinely done at the end of the general anesthesia and may also influence stomach motility in the further course.

DGE is regarded as a functional impairment of gastric motility and normal pyloric function, which has been supported by the observation that antecolic reconstruction of the duodeno-jejunal passage significantly lowered its incidence, probably due to the advantage of less chemical irritation by potential subclinical leakage of the pancreatic anastomosis within the first postoperative days. An additional anatomical modification by removing the pylorus could significantly enhance this effect. Of course, resection of the pylorus implies the risk of reflux symptoms in the long-term follow-up. Yet, this has not been proven and any long-term problems, for example gastric stump cancer after 10 to 15 years can be disregarded in pancreatic cancer patients. In patients undergoing PD for benign pathologies, however, these problems have to be taken into account and should be evaluated in long-term studies. A differential surgical approach for malignant and benign indications may be considered in case of clinically observed reflux-associated symptoms. Regarding perioperative morbidity and mortality, the available studies on prPD show comparable results underlining the feasibility of pylorus resection [9-11].

In conclusion, there is increasing evidence that prPD may be associated with a decreased rate of DGE. The present study as a large randomized trial based on the currently available literature is planned to confirm these findings, which may alter surgical procedures in PD.

## Trial status

### Recruiting

Enrolment of the first patient was performed in January 2013.

## Appendix

### escription of surgical and medical treatment

After confirmed respectability of the pancreatic process, patients are randomized either to the pylorus-preserving group or the pylorus-resecting group. When the pylorus is preserved, the transsection toward the specimen is carried out 1 cm distal to the pylorus by dividing the duodenum with a linear stapling and cutting device (GIA 75 mm, blue cartridge). In the pylorus resection group, this transsection is performed 1 cm proximal to the pylorus. Consequently, the stomach is divided by a linear stapling and cutting device (GIA 75 mm, green cartridge). The pylorus remains attached to the resection specimen. The further surgical procedure of resection and lymphadenectomy is carried out similarly in both groups as usual and according to local standards regarding the indication for the intervention.

After completion of the pancreatic and the bile duct anastomosis, the gastro-enteric passage is restored by either an end-to-side duodeno-jejunostomy in a two-layer fashion, using running sutures PDS 4-0 or by an end-to-side gastro-jejunostomy for which the same technique is used (two-layer, running sutures PDS 4-0). The anastomosis is located approximately 50 cm distal of the bile duct anastomosis in an antecolic position in both groups. A gastric tube is placed in the stomach without passing the anastomosis. This tube is removed after respiratory spontanization of the patient and removal of the endotracheal tube after the operation.

The expertise of the surgeon (board certified versus no certificate) who is performing the duodenal or gastric anastomosis has to be documented.

Duration of surgery (time from skin cut until closure (minutes)) and estimated blood loss (ml) will be documented.

### Permitted and not permitted medication(s)/treatment(s)

Standard medical treatment includes prokinetic medication with metoclopramide 3 × 10 mg/day either i.v. or orally and proton pump inhibition with pantoprazole 1 × 40 mg i.v. or per os. Antibiotic treatment includes the perioperative prophylaxis with mezlocillin and metronidazole or ciprofloxacin and metronidazole in case of an intolerance of penicillin-derived drugs. Additional postoperative care is performed according to the principles and standard of the department (pain treatment, fluid resuscitation).

Nasogastric tubes are removed immediately after the operation. Reinsertion is documented in the CRF with time of reinsertion and duration of the treatment.

Octreotide analogs can be used in cases with ‘high-risk’ pancreatic anastomoses according to the department guidelines. Antibiotics can be administered if necessary.

Erythromycin must not be used as an antibiotic agent. It is administered in case of delayed gastric emptying as a prokinetic drug for a three day period. Dosage, time of application and duration of the treatment must be recorded in the CRF.

Total parenteral nutrition is allowed in case of delayed gastric emptying. Dosage, time of application and duration of the treatment must be recorded in the CRF.

The occurrence of a lymphatic fistula that requires nihil per os treatment and total parenteral nutrition is a criterion that excludes the patient from the study as this interferes with the study endpoints.

Patients should receive the medical treatment according to the clinical situation. Any protocol violation has to be reported with a clear description in the CRF.

Endoscopic examination of the duodeno-jejunostomy or gastro-jejunostomy, respectively will be performed in case of DGE after three days of conservative treatment without success. Dilation of the anastomosis can be performed in case of suspected stenosis.

## Abbreviations

CRF: Case report form; DGE: Delayed gastric emptying; IPMN: Intraductal papillary mucinous neoplasia; IMBI: Institute for Medical Biometrics and Informatics; ISGPS: International Study Group for Pancreatic Surgery; PD: Partial duodeno-pancreatectomy; pp: Pylorus-preserving; pr: Pylorus-resecting; QoL: Quality of life; SOP: Standard operating procedure; RCT: Randomized controlled trial

## Competing interests

The authors declare that they have no competing interests.

## Authors’ contributions

TH and MWB conceived the study. TH, CDH, MKD, and OS drafted the study protocol. TB gave statistical advice. TH and LS drafted the manuscript. PK, WH, SF, JW, and MWB revised the manuscript. All authors read and approved the final manuscript.
